# Induced pluripotent stem cells representing Nakajo-Nishimura syndrome

**DOI:** 10.1186/s41232-019-0099-8

**Published:** 2019-05-23

**Authors:** Nobuo Kanazawa, Fumiko Honda-Ozaki, Megumu K. Saito

**Affiliations:** 1 Department of Dermatology, Wakayama Medical University, 811-1 Kimiidera, Wakayama, 641-0012 Japan; 2 Department of Clinical Application, Center for iPS cell Research and Application, Kyoto University, 53 Kawahara-cho, Shogoin, Sakyo-ku, Kyoto, 606-8507 Japan

**Keywords:** Nakajo-Nishimura syndrome, Proteasome-associated autoinflammatory syndromes, Immunoproteasome, PSMB8, Induced pluripotent stem cells, Monocytes

## Abstract

Nakajo-Nishimura syndrome is a proteasome-associated autoinflammatory syndrome with a distinct homozygous mutation in the *PSMB8* gene encoding an inducible β5i subunit of the immunoproteasome. Although it is considered that immunoproteasome dysfunction causes cellular stress and contributes to the production of inflammatory cytokines and chemokines, its detailed mechanism is still unknown. On the other hand, hereditary autoinflammatory diseases are considered as a good target for the analyses using induced pluripotent stem cells, whose differentiation systems to the innate immune cells such as neutrophils and monocytes have been established. Therefore, to elucidate the pathogenesis of Nakajo-Nishimura syndrome, we attempted in vitro disease modeling using patient-derived induced pluripotent stem cells. For analyses, isogenic control cells in which the responsible mutation was repaired and another pair of healthy embryonic stem cells and isogenic mutant cells in which the same mutation was introduced had also been prepared with genetic engineering. By comparing a pair of isogenic cells with the wild-type and the mutant *PSMB8* gene after differentiation into monocytes and immortalization to synchronize their differentiation stages, the reduction of immunoproteasome enzyme activity and increased cytokine and chemokine production in the mutant cells without stimulation or with interferon-γ plus tumor necrosis factor-α stimulation were observed, and therefore, the autoinflammatory phenotype was successfully reproduced. Decreased cytokine production was observed by the addition of antioxidants as well as inhibitors for Janus kinase and p38-mitogen-activated protein kinase. At the same time, the increased production of reactive oxygen species and phosphorylation of both signal transducers and activator of transcription 1 and p38-mitogen-activated protein kinase were detected without stimulation. Notably, an antioxidant specifically decreased the constitutive phosphorylation of signal transducers and activator of transcription 1. These results indicate the usefulness of a disease modeling using pluripotent stem cell-derived cells in clarification of the pathomechanism and discovery of new therapeutic drugs for Nakajo-Nishimura syndrome and related proteasome-associated autoinflammatory syndromes.

## Background

Nakajo-Nishimura syndrome (NNS) is a proteasome -associated autoinflammatory syndrome (PRAAS) with a distinct homozygous mutation in the *PSMB8* gene encoding an inducible β5i subunit of the immunoproteasome (iP). Although it is considered that iP dysfunction causes cellular stress and contributes to the production of inflammatory cytokines and chemokines, its detailed mechanism is still unknown. On the other hand, hereditary autoinflammatory diseases are considered as a good target for the analyses using induced pluripotent stem (iPS) cells, whose differentiation systems to the innate immune cells such as neutrophils and monocytes have been established. Therefore, to clarify the pathomechanism of NNS, we attempted in vitro disease modeling using patient-derived iPS cells and applied them to discover effective drugs.

## Main text

### Historical overview of NNS and related diseases

**Fig. 1:**
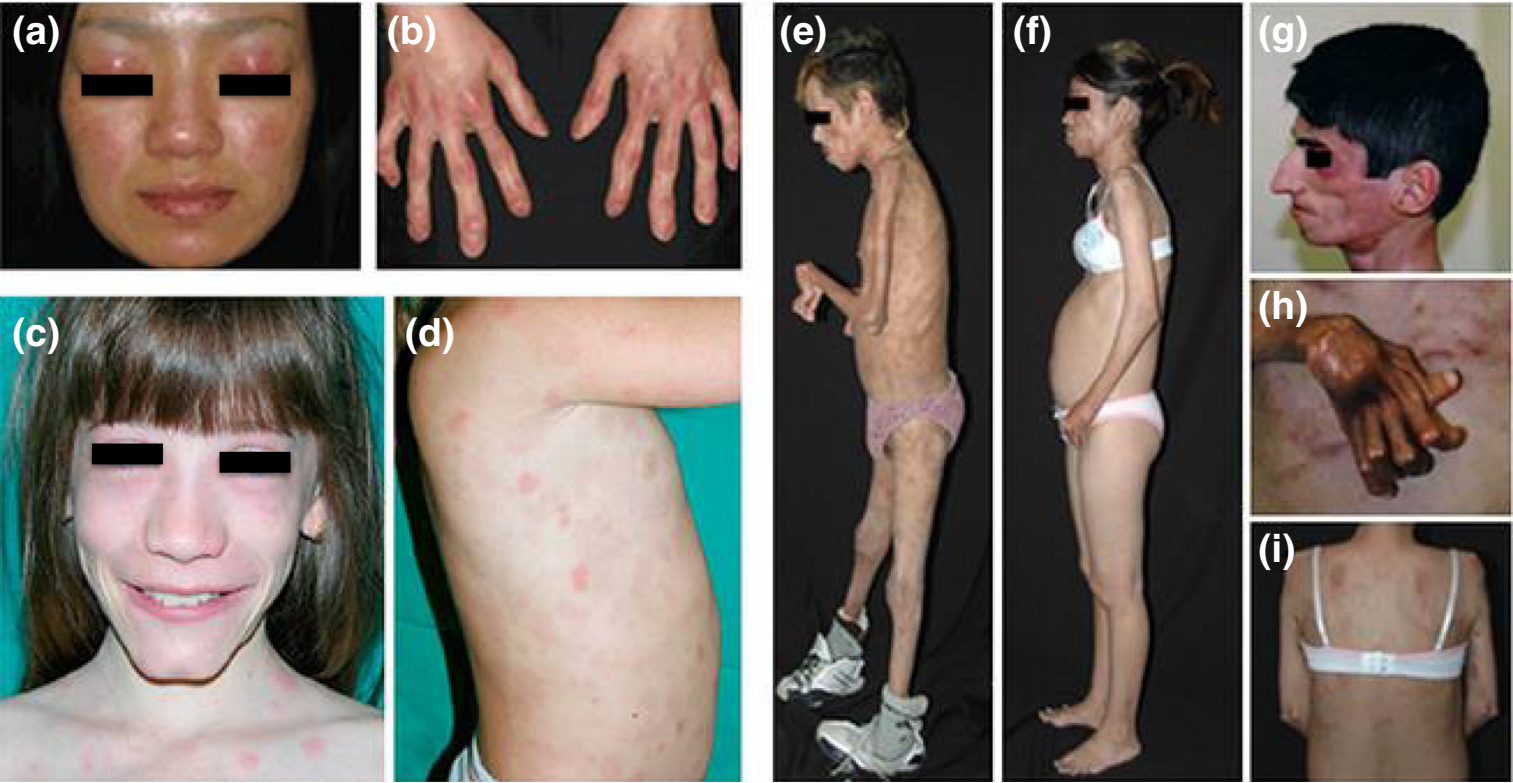
Clinical features of PRAASs. **a**, **b** NNS. **c**, **d** CANDLE syndrome. **e**–**i** JMP syndrome cases. (Reprinted with permission; McDermott et al*.* [13])

NNS belongs to the PRAASs caused by genetic defects of iP activities [1]. Historically, this disease was reported in Japanese as “secondary hypertrophic periostosis with pernio” for the first time in the world by dermatologists Nakajo and Nishimura et al. in 1939 and 1950, respectively [2, 3]. They described familial cases with parental consanguinity showing pernio-like or nodular erythema-like eruptions and progressive lipo-muscular atrophy. Although their cases and the following Japanese patients were collectively reviewed and introduced in English as “a syndrome with nodular erythema, elongated and thickened fingers, and emaciation” by dermatologists in 1985 and as “hereditary lipo-muscular atrophy with joint contracture, skin eruptions and hyper-γ-globulinemia” by neurologists in 1993, no such cases had been reported from any other countries than Japan until recent years [4, 5]. In 2010, Mexican and Portuguese cases were reported as “joint contractures, muscular atrophy, microcytic anemia, and panniculitis-associated lipodystrophy (JMP)” syndrome, which represented a similar but distinct entity from Japanese cases [6]. In the same year, similar cases were further reported as “chronic atypical neutrophilic dermatosis with lipodystrophy and elevated temperature (CANDLE)” syndrome by Spanish dermatologists and an autoinflammatory pathophysiology was suspected [7]. By the homozygosity mapping of familial cases, mutations in the *proteasome subunit beta type 8* (*PSMB8*) gene were successively identified in JMP syndrome, NNS, and CANDLE syndrome [8–10]. Japanese cases were also reported as “Japanese autoinflammatory syndrome with lipodystrophy (JASL),” and all Japanese NNS/JASL cases have the same mutation with a founder effect [11]. Now in Japan, NNS has been included in the officially registered intractable diseases and is diagnosed with a set of clinical features and genetic testing shown in Table 1 [1]. *PSMB8* encodes one of the iP-specific catalytic subunits, β5i. In JMP syndrome cases, the specifically impaired chymotrypsin-like activity was considered responsible [8]. In NNS cases, not only the impaired chymotrypsin-like activity but also the defective iP assembly leading to the impairment of all catalytic activities were observed [9]. No significant phenotype of *Psmb8*-null mice supports a hypothesis that not just a β5i loss-of-function but complexed iP dysfunction is responsible for these diseases [12]. Although the elevated serum levels of interferon (IFN)-inducible protein (IP)-10 and interleukin (IL)-6 were commonly observed in NNS and CANDLE cases, upregulated p38-mitogen-activated protein kinase (p38-MAPK) cascade and type I IFN signature were identified as characteristic in NNS cases and in CANDLE cases, respectively [9, 10]. Thus, any cellular stress with impaired iP activities are considered to cause excessive inflammatory reactions, and the underlying mechanisms are still largely unknown. The designation of PRAAS was first used when a case with compound heterozygous *PSMB8* mutations was reported, and then was further applied for the generic name of JMP syndrome, NNS/JASL, and CANDLE syndrome (Fig. 1) [13, 14]. Genetically, CANDLE/PRAAS cases were expanded to those with digenic mutations of iP-specific and nonspecific subunits, such as pairs of *PSMB8* plus *proteasome subunit alpha type 3* (*PSMA3*) encoding α7, *PSMB8* plus *PSMB4* encoding β7, and *PSMB9* encoding β1i plus *PSMB4* mutations, and to those with a heterozygous mutation in *proteasome maturation protein* (*POMP*) encoding a chaperon Ump1 [15]. In the database of Online Mendelian Inheritance in Man (OMIM), cases with recessive *PSMB8* and digenic mutations, those with a dominant *POMP* mutation, and those with recessive *PSMB4* or *PSMB9* and digenic mutations were registered as PRAAS1 (OMIM#256040), PRAAS2 (#618048), and PRAAS3 (#617591), respectively.

**Table 1:** Diagnostic criteria for Nakajo-Nishimura syndrome

1 Clinical manifestations	
1. Autosomal recessive inheritance (parental consanguinity and/or familial occurrence)	
2. Pernio-like purplish rash in hands and feet (appearing in winter since infancy)	
3. Haunting nodular erythema with infiltration and induration (sometimes circumscribed)	
4. Repetitive spiking fever (periodic, not necessarily)	
5. Long clubbed fingers and toes with joint contractures	
6. Progressive partial lipomuscular atrophy and emaciation (marked in the upper part of body)	
7. Hepatosplenomegaly	
8. Basal ganglia calcification	
2 *PSMB8* gene analysis	
Flowchart for diagnosis	
1) In case at least five out of eight clinical manifestations are positive and other disorders can be excluded, diagnosis as NNS can be clinically defined. If not, only suspected. When NNS is clinically defined or suspected, the *PSMB8* gene is analyzed.	
2) *Definite*: Clinically defined or suspected, and disease-associated *PSMB8* mutation detected in both alleles	
3) *Probable*: Clinically defined, but no disease-associated *PSMB8* mutation detected in both alleles

### The proteasome system

**Fig. 2:**
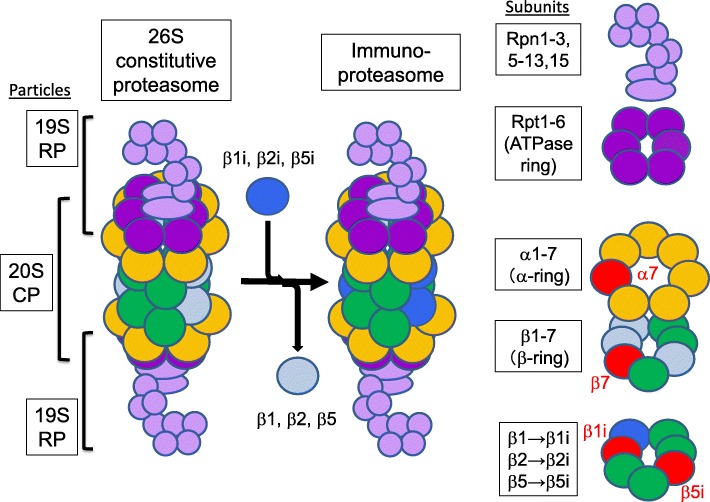
Scheme of the proteasome complex. Subunits with mutations causing PRAASs were shown in red

The *PSMB8* gene encodes an inducible β5i subunit of the proteasome, a huge enzyme complex responsible for the non-lysosomal proteolysis system in the cell. Seven α subunits (α1–7) and seven β subunits (β1–7) are arranged side by side to form the α- and β-ring, respectively, and they are combined up and down to form the half proteasome. Two of the half proteasomes are combined up and down (α-β-β-α-rings) to form the 20S core particle (CP), and a combination of 19S regulatory particle (RP) above and below 20S is called the constitutive 26S proteasome (Fig. 2) [16]. The 19S RP recognizes unnecessary and deleterious proteins specifically through their polyubiquitin label and guides these proteins to the 20S CP, which degrades them by the catalytic subunits β1, β2, and β5. However, the role of this selective degradation system called the ubiquitin-proteasome system is not limited to the removal of unnecessary and deleterious proteins, but involved in various cellular functions such as cell cycle and signal transduction. Furthermore, the iP, in which the catalytic β1, β2, and β5 subunits are replaced with highly active β1i, β2i, and β5i subunits of the inducible type, is constantly expressed in immunocompetent cells, and at the time of inflammation, it is also induced in other somatic cells by inflammatory stimuli such as IFN-γ and tumor necrosis factor-α (TNF-α). The iP shows not only the higher enzymatic activities, but also the more efficient production of antigenic peptides presented on major histocompatibility complex (MHC) class I to activate CD8 T cells, which contributes to the activation of acquired immunity. Thus, the iP is considered to work for linking the innate and the acquired immunity. However, MHC class I expression has not been affected in NNS patients’ cells (unpublished data) and no apparent immunodeficiency with severe and/or frequent infection has been observed in the patients. Rather, some cases with NNS show a mild elevation of serum autoantibodies during the disease course, which might be associated with type I interferonopathy, and NNS/PRAASs are considered to be located in close contact with autoimmune diseases [1, 17].

### The iPS cells

**Fig. 3:**
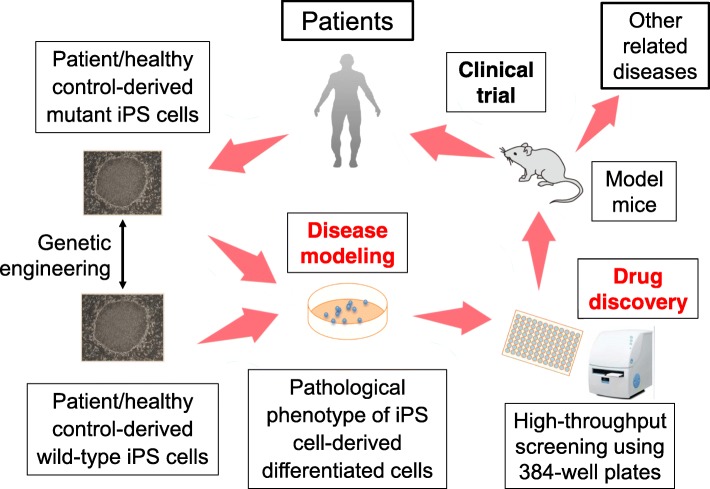
Strategy of disease modeling and drug discovery using iPS cells. If a major aspect of the disease phenotype is reproduced in vitro using a genetically engineered pair of mutant and wild-type iPS cells (disease modeling), a compound which can recover the phenotype is searched with high-throughput screening and provided for clinical trial following its application to any model mice in vivo (drug discovery)

The iPS cells represent a kind of pluripotent stem (PS) cells established by Yamanaka and colleagues [18, 19]. Among PS cells which are defined by two capacities, (1) the pluripotency as an ability to differentiate into all cells and tissues constituting an individual and (2) the self-replicating ability iPS cells are highly beneficial because they can be induced from somatic cells. Established iPS cells contain the same genome as the individual who provided the original somatic cells. Therefore, by providing somatic cells such as blood cells and skin fibroblasts from patients with hereditary diseases, generated iPS cells share the patient’s genetic background. Although the cell type responsible for the pathogenesis would be different depending on the patient’s disease, it is possible to reproduce a part of the patient’s disease condition in vitro by differentiating these iPS cells to the disease-responsible cells and observing their phenotype. As autoinflammatory diseases are mostly inheritable and the differentiation systems from iPS cells to their major responsible innate immune cells such as neutrophils and monocytes have been established, they are considered as a disease group which is applicable for the analyses using iPS cells. Furthermore, since autoinflammatory diseases represent a relatively new concept and are still expanding with discoveries of novel responsible mutations, establishment of their diagnostic and therapeutic technologies and elucidation of their pathomechanisms are still required. Therefore, a disease modeling using iPS cells is expected to be useful for resolving these issues. Especially, by establishing in vitro high-throughput selection system, discovering drugs which improve the disease-responsible phenotype modeled with iPS cells will be possible (Fig. 3). Therefore, here, we attempted to apply this system for clarifying the pathomechanism of NNS and discovering effective drugs [20].

### Generation of NNS-type mutant iPS cell-derived monocytes and control cells

First, reprogramming primary fibroblasts prepared from three NNS patients with the homozygous *PSMB8* c.602G > T causing p.G201 V mutation was performed after obtaining written informed consents by introducing the so-called Yamanaka four factors, OCT3/4, SOX2, KLF4, and cMYC, using retroviral vectors. Although the proteasome complex is known to influence the induction, maintenance, and termination of self-renewal and pluripotency of PS cells, generated iPS cell clones showed characteristic features of PS cells in morphology, expression of specific markers, and ability to form teratoma composed of all three germ layers [21, 22].

A representative one clone (NNS1-1 [MT]) prepared from patient 1 was selected, and an isogenic clone (NNS1-1 [WT]) was created using the CRISPR/Cas9 system. Similarly, another mutant PS cell clone (KhES-1 [MT]) was prepared by introducing the homozygous *PSMB8* c.602G > T mutation into a healthy human embryonic stem (ES) cell clone (KhES-1 [WT]). The efficacy of genome editing was comparable between NNS1-1 [MT] and KhES-1 [WT] clones, and no impairment of the chymotrypsin-like activity was observed in [MT] PS cells compared with the control [WT] PS cells, even after stimulation with IFN-γ and TNF-α.

Generated two isogenic pairs of PS cell clones (NNS-1 [WT] and NNS-1 [MT], KhES-1 [WT] and KhES-1 [MT]) were differentiated into CD14^+^ monocytes at an efficiency of about 90% according to the already established protocol [23]. No difference between differentiated [WT] and the corresponding [MT] cells was still observed in the monocytic morphology and the expression levels of CD14 and CD33. Thus, the NNS-type *PSMB8* mutation showed no apparent effect on both the generation of iPS cells and the in vitro hematopoiesis with monocytic differentiation. This result is clinically compatible to the patients but is incompatible to the case of adipocyte differentiation, which was reportedly impaired by the NNS-type *PSMB8* mutation [24]. Differentiated monocytic cells were further immortalized by the introduction of MDM2, BMI1, and cMYC, using a lentiviral vector to synchronize their differentiation stages and to obtain an unlimited number of cells with easier handling.

### In vitro disease modeling using NNS-type mutant iPS cell-derived monocytes

Using two pairs of PS cell-derived monocytic cell lines (PSC-MLs), significant impairment of the chymotrypsin-like activity was commonly observed in [MT] PSC-MLs compared with the control [WT] PSC-MLs after stimulation with IFN-γ and TNF-α. Although not significant, the same tendency was observed without stimulation. Notably, the degradation of not the β5-specific but the β5i-specific substrate was significantly impaired in [MT] PSC-MLs compared with the [WT] counterpart after stimulation with IFN-γ and TNF-α. Furthermore, the impaired maturation of β5i and the accumulation of proteins were also observed in [MT] PSC-MLs compared with the [WT] counterpart before and after stimulation with IFN-γ and TNF-α by western blotting. These results are compatible to those using the NNS patient’s immortalized B cells [9].

Regarding the cytokine and chemokine production, significantly increased secretion of IL-6 and monocyte chemoattractant protein (MCP)-1 was commonly observed in [MT] PSC-MLs compared with the control [WT] PSC-MLs even without stimulation and after stimulation with IFN-γ and TNF-α. In contrast, significantly increased secretion of IP-10 was only observed in NNS-1-derived [MT] MLs compared with the [WT] counterpart after stimulation with IFN-γ and TNF-α. Rather, stimulation with only IFN-α or IFN-γ specifically induced IP-10 secretion in both [MT] PSC-MLs compared with the [WT] counterpart, to a much higher amount when stimulated by IFN-γ. Neither of IFN-α or IFN-β secretion was observed in any of [WT] and [MT] PSC-MLs after stimulation with IFN-γ plus TNF-α or with lipopolysaccharide (LPS). The increased secretion of IL-6 in LPS plus adenosine triphosphate-stimulated and IP-10 in IFN-γ-stimulated NNS patients’ peripheral blood mononuclear cells (PBMC) was also observed, compared with those in healthy control PBMC. Moreover, the high serum levels of IL-6, MCP-1, and IP-10 were also characteristic in NNS patients [9]. Thus, at least one aspect of NNS was successfully reproduced in vitro by these PSC-MLs.

Interestingly, a principle component analysis of the result of whole RNA sequencing revealed a different grouping of [WT] and [MT] PSC-MLs even without stimulation, indicating that the NNS-type *PSMB8* mutation provides a kind of “priming” for monocytes in the steady state. A gene set enrichment analysis further revealed that IFN-γ plus TNF-α-stimulated [MT] PSC-MLs showed significantly higher expression of the genes for IFN-γ response, IFN-α response, and TNF-α signaling.

### Effects of various anti-inflammatory compounds on NNS-type mutant iPS cell-derived monocytes

**Fig. 4:**
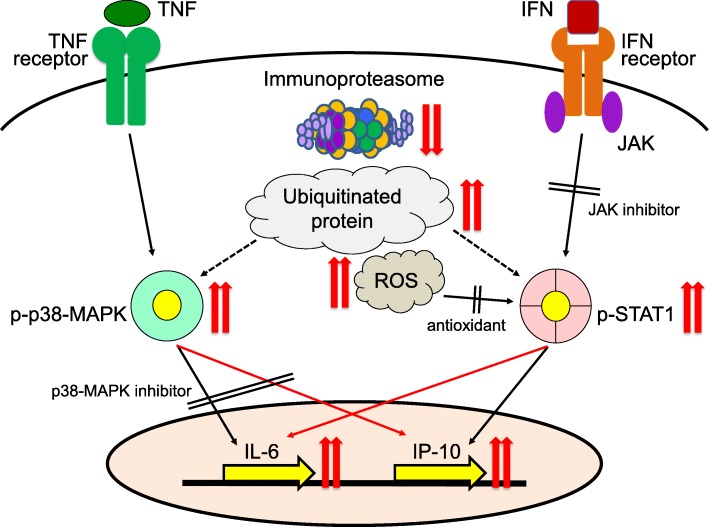
Scheme of the autoinflammatory pathomechanism in NNS. In NNS-type-mutated PSC-MLs, the ROS productions as well as the accumulation of ubiquitinated proteins are constitutively induced by the iP dysfunction. The STAT1 phosphorylation contributed by ROS, accompanied by the p38-MAPK phosphorylation, provides the “priming” inflammatory signal. After IFN-γ plus TNF-α-stimulation, any of a pan-JAK inhibitor, a p38-MAPK inhibitor, and antioxidants significantly reduced both IL-6 and IP-10 secretion, suggesting a pathological crosstalk between the IFN-JAK-STAT-IP-10 pathway and the TNF-α-p38-MAPK-IL-6 pathway

The effects of clinically relevant anti-inflammatory compounds on cytokine production by IFN-γ plus TNF-α-stimulated [MT] PSC-MLs were estimated. Dexamethasone, which can inhibit the activation of nuclear factor-κB and MAPK pathways, significantly reduced IL-6 and MCP-1 secretion in some of the stimulated PSC-MLs, whereas it had no effect on IP-10 secretion. In contrast, both of a pan-Janus kinase (JAK) inhibitor and a p38-MAPK inhibitor significantly reduced all IL-6, MCP-1, and IP-10 secretion in any of the stimulated PSC-MLs in a dose-dependent manner. This result suggests a possibility that any pathological crosstalk is present between the IFN-JAK-signal transducers and activator of transcription (STAT)-IP-10 pathway and the TNF-α-p38-MAPK-IL-6 pathway in NNS-type [MT] PSC-MLs (Fig. 4).

### Characterization of the priming in NNS-type mutant iPS cell-derived monocytes

Impaired iP activity causes an accumulation of ubiquitinated proteins and might cause some cellular stresses. A proteasome inhibitor bortezomib reportedly causes damage to the mitochondria, leading the cells to increase the production of reactive oxygen species (ROS) [25]. Mitochondrial oxidative stress has been shown to cause an increased production of inflammatory cytokines and chemokines [26]. Therefore, the cellular level of ROS was examined in PSC-MLs and a tendency of increased production of ROS was commonly observed in [MT] PSC-MLs compared with the control [WT] PSC-MLs after stimulation with IFN-γ and TNF-α, while its significant increase was rather observed in the steady state without stimulation. Furthermore, antioxidants glutathione-SH and *N*-acethyl-cysteine (NAC) significantly reduced all IL-6, MCP-1, and IP-10 secretion in most of the unstimulated and IFN-γ plus TNF-α-stimulated PSC-MLs in a dose-dependent manner.

By western blotting, phosphorylated STAT1 (p-STAT1) and p-P38-MAPK were already detected more strongly in [MT] PSC-MLs than in [WT] counterpart without stimulation. Interestingly, NAC reduced the p-STAT1 level without changing the p-P38-MAPK level in unstimulated and even IFN-γ plus TNF-α-stimulated [MT] PSC-MLs. As a tendency of increased production of mitochondrial ROS was also commonly observed and no effect of a nicotinamide adenine dinucleotide phosphate oxidase (NOX) inhibitor on either of the increased ROS production or the cytokine production was observed in [MT] PSC-MLs compared with the [WT] counterpart before and after IFN-γ plus TNF-α stimulation, it is suggested that the primary source of the cellular ROS was mitochondria rather than NOX in [MT] PSC-MLs. Thus, NNS-type [MT] PSC-MLs are already primed in the steady state, characterized by the overproduction of mitochondria-derived ROS which leads to the higher p-STAT1 level and by the higher p-p38-MAPK level. Then, IFN-γ plus TNF-α stimulation causes overactivation of JAK/STAT and p38-MAPK pathways through the higher basal p-STAT1 and p-p38-MAPK levels (Fig. 4).

## Conclusions

Modeling the autoinflammatory phenotype of NNS has been performed successfully using PS (patient-derived iPS and healthy ES) cell-derived monocytes. A ROS-mediated “priming” inflammatory signal and a pathological crosstalk between the JAK-STAT and the p38-MAPK pathways by the NNS-type *PSMB8* mutation have newly been shown (Fig. 4). In addition, therapeutic effects of antioxidants as well as pan-JAK inhibitor and p38-MAPK inhibitor, which were better than that of dexamethasone, on the increased cytokine and chemokine production of the NNS-type PS cell-derived monocytes were revealed. These results indicate the usefulness of such a disease modeling using PS cell-derived cells in the clarification of the disease pathomechanism and discovery of new therapeutic drugs. Regarding NNS and related PRAASs, clarification of the common and/or distinct points among these diseases and development of the commonly and/or specifically effective drugs are expected.

## Abbreviations

CANDLE: Chronic atypical neutrophilic dermatitis with lipodystrophy and elevated temperature
CP: Core particle
ES: Embryonic stem
IFN: Interferon
IL: Interleukin
iP: Immunoproteasome
IP: Interferon-inducible protein
iPS: Induced pluripotent stem
JAK: Janus kinase
JASL: Japanese autoinflammatory syndrome with lipodystrophy
JMP: Joint contractures, muscular atrophy, microcytic anemia, and panniculitis-associated lipodystrophy
LPS: Lipopolysaccharide
MAPK: Mitogen-activated protein kinase
MCP: Monocyte chemoattractant protein
MHC: Major histocompatibility complex
NAC: *N*-Acetyl-cysteine
NNS: Nakajo-Nishimura syndrome
NOX: Nicotinamide adenine dinucleotide phosphate oxidase
OMIM: Online Mendelian Inheritance in Man
p-: Phosphorylated
PBMC: Peripheral blood mononuclear cells
POMP: Proteasome maturation protein
PRAAS: Proteasome-associated autoinflammatory syndrome
PS: Pluripotent stem
PSC-MLs: Pluripotent stem cell-derived monocytic cell lines
PSMA: Proteasome subunit alpha type
PSMB: Proteasome subunit beta type
ROS: Reactive oxygen species
RP: Regulatory particle
STAT: Signal transducers and activator of transcription
TNF: Tumor necrosis factor
